# Methods used in the Lives Saved Tool (LiST)

**DOI:** 10.1186/1471-2458-11-S3-S32

**Published:** 2011-04-13

**Authors:** William Winfrey, Robert McKinnon, John Stover

**Affiliations:** 1 Futures Institute, 41-A New London Turnpike, Glastonbury, Connecticut, 06033, USA

## Abstract

**Background:**

Choosing an optimum set of child health interventions for maximum mortality impact is important within resource poor policy environments. The Lives Saved Tool (LiST) is a computer model that estimates the mortality and stillbirth impact of scaling up proven maternal and child health interventions. This paper will describe the methods used to estimate the impact of scaling up interventions on neonatal and child mortality.

**Model structure and assumptions:**

LiST estimates mortality impact via five age bands 0 months, 1-5 months, 6-11 months, 12-23 months and 24 to 59 months. For each of these age bands reductions in cause specific mortality are estimated. Nutrition interventions can impact either nutritional statuses or directly impact mortality. In the former case, LiST acts as a cohort model where current nutritional statuses such as stunting impact the probability of stunting as the cohort ages. LiST links with a demographic projections model (DemProj) to estimate the deaths and deaths averted due to the reductions in mortality rates.

**Using LiST:**

LiST can be downloaded at http://www.jhsph.edu/dept/ih/IIP/list/ where simple instructions are available for installation. LiST includes default values for coverage and effectiveness for many less developed countries obtained from credible sources.

**Conclusions:**

The development of LiST is a continuing process. Via technical inputs from the Child Health Epidemiological Group, effectiveness values are updated, interventions are adopted and new features added.

## Background

Human and financial resources for expansion of health services are limited. Therefore resources should be directed toward expanding availability and use of services that have the greatest health impact. Health policy makers and program managers require a tool that allows them to assess the differential mortality impact of a comprehensive set of maternal and child health interventions. Previously developed tools are either narrowly focused on a single set of interventions or calculate impacts without a rigorous demographic or epidemiological framework. LiST overcomes these limitations by allowing the simultaneous projection of health impacts for many maternal and child health interventions. This is primarily done by linking LiST as an additional module to the Spectrum suite of projection models that includes a demographic projection model, an HIV/AIDS projection model and a model for assessing the demographic impacts of family planning programs. Spectrum and the integration of LiST with other modules of SPECTRUM are described in Stover et al.[1]

LiST is a computer projection model used to estimate the number of deaths that can be averted as a result of scaling up effective maternal and child health interventions in developing countries. The work presented here is an instantiation of the work performed by the Child Health Epidemiology Reference Group (CHERG) for WHO and UNICEF. The model and approach used in LiST are drawn from the modeling in a series of papers in Lancet, including the Child Survival Series [2], the Neonatal Series [3] and the Maternal and Child Undernutrition series.[4] A complete description of the uses of LiST and background on its creation including, expert technical inputs can be found in Boschi-Pinto et al.[5]

This paper describes the technical details of the model for scaling up non-AIDS interventions for preventing deaths and improving health status among children under the age of five. While LiST also estimates the impact of interventions on maternal mortality and still births, these outputs will not be discussed in detail here. LiST estimates the direct impact of interventions on mortality as well as the indirect effects of interventions that act through intermediate outcomes, such as stunting. The model projects mortality rates including the neonatal mortality rate, the infant mortality rate and the under-five mortality rate. With the help of the demographic engine in Spectrum, LiST also calculates the numbers of deaths disaggregated by cause of death and by age band. Deaths averted are also disaggregated by cause of death, age band and intervention. The mortality impact of scaling up PMTCT, Cotrimoxazole and ART for children is independently calculated within the AIDS Impact Module (AIM) of SPECTRUM. Reductions in AIDS mortality are calculated with AIM because the mortality from AIDS is contingent upon the prevalence of AIDS among adults. AIM contains an epidemiological model for AIDS among the entire population in contrast to LiST which is directed uniquely at child mortality.

LiST requires three sets of inputs: a) intervention coverages that can be scaled up from a projection baseline or from the first year of an intervention program; b) measures of health status (e.g., levels of risk factors and population exposures, and baseline cause-specific mortality estimates); and c) estimates of intervention effectiveness. LiST estimates mortality reductions from a projection baseline. The projection baseline is the most recent point in time where a complete set of baseline intervention coverages and mortality are known.

LiST is loaded with default baseline coverage values, measures of health status, levels of risk factors, population exposures and cause of death data for more than 80 countries. The user is encouraged to choose his/her own scale up trajectories of coverage for all interventions of interest. Occasionally the user will have health status or mortality information that s/he believes is more up to date than that provided within LiST. In these cases s/he may want to update this information within LiST for his/her country. There is also a complete set of intervention effectiveness estimates in LiST. These effectiveness values are based on reviews performed by the Child Health Epidemiological Group of WHO and UNICEF. Examples of these reviews can be found in this issue as well as in earlier publications. [6].

This paper describes how the model is constructed and presents key equations used to estimate the impact of health interventions on child mortality.

## Model structure, assumptions and formulae

### Outline of basic structure

LiST is a partial cohort model which follows children through five age bands from birth to five years of age. Mortality rates and causes of death are described for neonates (under 1 month of age), children 1-59 months of age, and women giving birth (Table 1). From this information the model determines the number of deaths by cause each year. Current mortality rates can be reduced through increased coverage of any of 77 health interventions organized by the time period when the interventions are implemented: periconceptual, pregnancy, childbirth, breastfeeding promotion, preventive after birth, vaccines and curative after birth (Additional File 1).

**Table 1:** Age bands and causes of death modeled in LiST.

Age band	Causes of death acting during age band
Birth to 0.9 months	Birth asphyxia, prematurity, sepsis/pneumonia, congenital anomalies, tetanus, diarrhoea, all other causes of death

1 – 5.9 months	Diarrhoea, pneumonia, meningitis, measles, malaria, pertussis, injury, AIDS, all other causes of death
6 - 11.9 months	
12 – 23.9 months	
24 – 59.9 months	

Women giving birth (maternal mortality)	Antepartum hemorrhage, postpartum hemorrhage, hypertensive diseases of pregnancy, sepsis, abortion, obstructed labor, ectopic pregnancy, malaria, other causes of death

Women giving birth (still births)	Antepartum, intrapartum

Interventions may have a direct effect on a specific cause of death or an indirect effect. LiST does not assume that secular trends of mortality decline will continue. Only intervention scale up will cause a change in mortality rates. Increasing the coverage of any given intervention may cause a reduction in multiple causes of death. For example, zinc for prevention will decrease both diarrhoea mortality and pneumonia directly. Zinc for prevention will also decrease stunting which has downstream impacts on diarrhoea, malaria, measles and pneumonia mortality. Indirect effects work though intermediate characteristics or risk factors including the proportion of children born with intra-uterine growth restriction (IUGR); nutritional status of children, as measured by height-for-age z-score (HAZ) and weight-for-height z-score (WHZ); and diarrhoea incidence. These indirect characteristics or risk factors impact mortality rates. Outcomes are calculated for mortality rates (neonatal, infant, child and maternal mortality), numbers of still births, numbers of deaths by cause of death, and deaths averted by intervention and cause of death.

### Calculating the direct effects of interventions on mortality

Direct effects are calculated when a particular intervention directly reduces mortality from a specific cause. For example, expanding the coverage of oral rehydration salts (ORS) directly reduces mortality from diarrhoea. The reduction in mortality caused by a specific intervention is calculated from the increased coverage of the intervention multiplied by the effectiveness of the intervention in reducing mortality. This crude estimate is adjusted for the impact of current coverage that is already incorporated in current mortality rates (by dividing the crude impact by the potential mortality reduction remaining). Since some interventions may not act against all mechanisms of mortality, the impact is further adjusted for the proportion of cause-specific mortality that is susceptible to that intervention, called the Affected Fraction. For example, ORS is effective against some forms of diarrhoea but not all. Thus the affected fraction of ORS is 0.95, indicating that 5% of diarrhoea deaths are due to mechanisms not affected by ORS.

In detail, the proportional reduction *R* in mortality from cause of death *j* for children in age band *a* caused by intervention *i* at time *t* (*R_i_*_,_*_j_*_,_*_a_*_,_*_t_*) is a function of the effectiveness of the intervention *I_i_*_,_*_j_*_,_*_a_*, the increase in the coverage of intervention *I* (*C_i_*_,_*_a_*_,_*_t_ – C_i_*_,_*_a_*_,_*_0_*) and the affected fraction (*AF_i_*_,_*_j_*_,_*_a_*) adjusted for the unrealized potential impact (*1 – I_i_*_,_*_j_*_,_*_a_*_,_*_0_ x C_i_*_,_*_a_*_,_*_0_*).

R_i,j,a,t_ = [I_i,j,a_ x (C_i,a,t_ – C_i,a,0_) / ( 1 – I_i,j,a,0_ x C_i,a,0_)] x AF_i,j,a_ (1)

For example, suppose that the coverage of ORS increased from 25 percent to 50 percent and that the effectiveness of ORS was 0.93 and the fraction of diarrhoea deaths that could be prevented by ORS was 0.95. If ORS were the only intervention then the percent reduction in mortality would be: [0.930 * (0.50 – 0.25)] / (1 – 0.93*0.25) * 0.95 = 0.288 or a 28.8 percent reduction in diarrhoea mortality.

When more than one intervention is scaled up LiST first calculates the mortality reduction for each intervention in isolation, as if it were the only intervention implemented. The total mortality reduction for a package of interventions is calculated by sequencing the impact calculations such that the first intervention acts on the current level of mortality, while the second, third and subsequent interventions act on the remaining mortality, after the effects of the previous interventions have been removed. In mathematical terms, the total impact of all interventions (*R_j_*_,_*_a_*_,_*_t_*) is the product of the impact of each intervention on the remaining mortality:

R_j,a,t_ = 1 – (1 - R_1,j,a,t_ ) * (1 – R_2,j,a,t_) * (1 – R_3,j,a,t_) * (1 – R_4,j,a,t_) … (2)

The ordering of the interventions in this calculation is irrelevant because the total impact does not depend on the order. On the other hand, attributing the share of the impact to a specific intervention depends on the timing of the intervention. This is discussed below.

**Figure 1:**
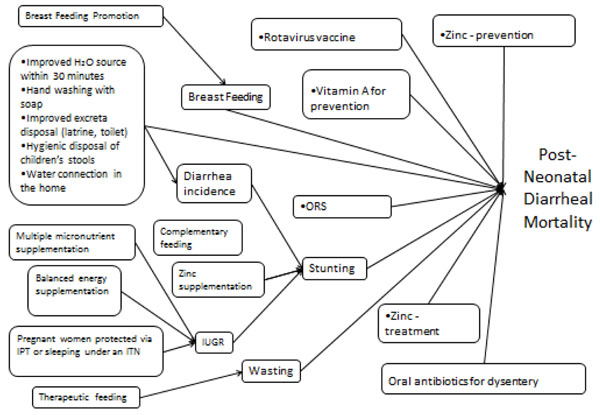
LiST calculation of postneonatal diarrhoea deaths in children aged 1 month to 59 months

In LiST there are currently 11 interventions that impact diarrhea mortality (see figure 1). As an example assume that two interventions are being scaled up to reduce diarrhoea mortality: ORS and zinc tablets for treatment. If ORS reduces diarrhoea mortality by 28.8 percent and Zinc reduces mortality by 25.0 percent then the combined reduction in mortality would be 1 – (1 -0.288) x (1- 0.25) = 0.466 or a 46.6 percent reduction.

In the simplest case, the mortality reduction attributed to each individual intervention (*R’*) is determined as the total mortality reduction multiplied by the full impact of the intervention when calculated in isolation divided by the sum of the full impacts of all interventions.

R’_i,j,s,t_ = R_j,s,t_ x R_i,j,s,t_ / ∑_i_ R_i,j,s,t_ (3)

Following the example from above, the shares of mortality reduction for cause j allocated to ORS and zinc for treatment respectively would be: 0.288 / (0.288 + 0.250) = 0.535 for ORS and 0.250 / (0.288 + 0.250) = 0.465 for Zinc.

A complicating factor is that interventions are characterized by the timing of their impact. Peri-conceptual interventions are first in line, followed in turn by pregnancy related interventions, childbirth interventions, vaccines, “preventive after birth” interventions and finally “curative after birth” interventions. The interventions whose timing is first receive credit for mortality reductions as if they were working on the entire burden of cause specific mortality. The subsequent sequential interventions receive credit as if they were only acting on the cause specific mortality that remained after the upstream interventions have acted.

Following the previous example, suppose that coverage of rotavirus vaccine was scaled up in addition to ORS and zinc for treatment. Rotavirus vaccine is a preventive intervention and acts on mortality before ORS and zinc for treatment. Assume that the by equation (1) the independent impact of the rotavirus vaccine would be 30.6. Using the equation (2) the total impact would be 62.9 percent. Since rotavirus is the only vaccine and it acts before the other interventions (both curative) it receives full credit in its share. Rotavirus share of the mortality reduction is therefore 0.306/.629 or 48.6%. ORS and zinc for treatment then share the mortality reduction that remains. Applying the shares that were calculated before ORS would have a share of (0.629 – 0.306)/0.629 x 0.535 = 0.173 or 27.5% and zinc for treatment would have a share of (0.629 – 0.306) / 0.629 x 0.465 = 0.239 or 23.9%.

The reduction in all cause mortality is simply the reduction from all interventions acting on a single cause of death multiplied by the proportion of all deaths due to that cause. Thus, if diarrhoea mortality is reduced by 46% due to ORS and zinc for treatment, and diarrhoea is responsible for 20% of all deaths in that age band, then the reduction in all cause mortality would be 0.46 x 0.20 = 0.09. Reductions in mortality from other causes of death are applied similarly to determine the total reduction in all causes of death.

The number of deaths averted is determined by applying the reduction in the overall mortality rates to the number of children in each age band. The number of children is provided by the demographic projection module in Spectrum. The number of deaths averted by an intervention is calculated as the product of the total number of deaths averted and the proportional contribution of each intervention. Because an intervention frequently has an impact on several causes of death, LiST sums the deaths averted by an intervention across the several causes of death to determine the total impact of each intervention.

Because a demographic model lies behind LiST there are several spillover effects. For example, a reduction in neonatal mortality will result in an increased number of children exposed to mortality after the neonatal period. In the absence of mortality reduction measures for children aged 1-59 months there would be more deaths to older children than otherwise would have occurred. Likewise, problems in the neonatal period can have an effect on the risk of mortality during the period 1-59 months via inter-uterine growth retardation. Children with IUGR have a higher rate of mortality in the neonatal period but also a higher risk of of stunting among children aged 1 -59 months, leading to reduced mortality among the older children.

#### Herd effect of vaccines and bednets

Estimates of the impact of vaccinations and/or bednets may include a herd effect caused by the disruption of transmission. The herd effect occurs when some children who are not themselves vaccinated or sleeping under a bednet are protected from infection due to the reduced exposure to infection caused the large percentage of other children who are vaccinated or sleeping under bednets. These calculations require an assumption about the protection received by children not receiving the intervention as a function of the overall population coverage. The additional reduction in mortality due to the herd effect (H_i,j,a,t_) is calculated as the difference in the herd effect (HE_i,j,a,t_) at the current coverage and the baseline coverage (HE_i,j,a,0_), adjusted for the effect at the baseline coverage.

H_i,j,a,t_ = (HE_i,j,a,t_ – HE_i,j,a,0_) / ( 1 – HE_i,j,a,0_) (4)

The herd effect of the vaccine acts in tandem with its direct protective effect to produce the total impact on mortality when the herd effect is present (hR).

hR_i,j,a,t_ = R_i,j,a,t_ + H_i,j,a,t_ x (1 - R_i,j,a,t_) (5)

For the measles vaccine a herd effect of 1.00 exists for coverage that exceeds 95 percent. For coverage between 90 percent and 95 percent the herd effect is the interpolated value between 0.00 and 1.00. For values of coverage below 90 percent the herd effect is 0.00. The effectiveness of the measles vaccine is 0.850. If baseline coverage of the measles vaccine was 75 percent and the target coverage was 97 percent then the additional reduction in mortality due to the herd effect would be (1.00 – 0.00)/(1.00 – 0.00) = 1.00. Using equation (1) R_i,j,a,t_ would be 0.516. By equation (5) the total reduction in mortality due to the measles vaccine would be 0.516 + 1.00 x ( 1 – 0.516) = 1.00, a 100% reduction in mortality.

### Calculating the effects of interventions on intermediate health outcomes

The previous discussion describes the procedures used within LiST for calculating the effects of interventions that directly reduce mortality from specific causes of death. Other interventions, such as nutrition, affect mortality indirectly through intermediate health outcomes such as stunting, which in turn affect mortality. For example, zinc supplementation for prevention may affect stunting which in turn may affect mortality from diarrhoea.

Zinc supplementation for prevention → stunting → diarrhoea mortality

The following subsections describe the approach used to capture these indirect effects.

#### Indirect effect

LiST includes four intermediate health outcomes or risk factors that affect mortality: stunting, wasting, intrauterine growth restriction (IUGR) and diarrhoea incidence (which actually affects another intermediate outcome, stunting). Breastfeeding is a special intermediate outcome that is discussed below.

#### Stunting and IUGR

**Figure 2:**
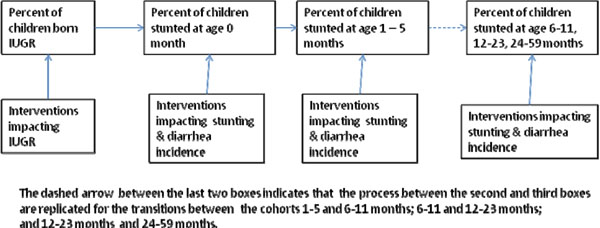
Schematic of the process behind the calculation of stunting

Interventions impacting stunting are modeled via cohorts such that the nutritional status of a child in a given cohort will continue to impact nutritional status as the child ages. In other words a child that is born with IUGR will be more likely to be stunted in the first month of life and children who are stunted at the end of an age band will be more likely to be stunted as they age into the next age band. Figure 2 is a summary schematic of the approach to modeling.

The impact of interventions and conditions that impact stunting are modeled as odds ratios. The odds of stunting is the probability of being stunted divided by the probability of not being stunted: Odds = P / [1 - P]. The odds ratio formulation was chosen to assure that the risk of stunting would never exceed 1.00. The odds ratio compares the odds of stunting for children in two different statuses. For example the odds ratio of being stunted relative to not being stunted for children who do not receive zinc supplements for prevention versus that for children who are zinc supplemented has a default value 1.180. This means that the odds of being stunted for a child who has not received zinc supplements for prevention are 18 percent higher than the odds of being stunted for a child who has received zinc supplements.

Continuing with the zinc example, if a child moves from a state of not being zinc supplemented to a state of being zinc supplemented her odds of being stunted decline. The risk of a child who is zinc supplemented is related to the risk for children who are not zinc supplemented by equation (6). This equation is derived from the definition of an odds ratio.

P_k,a_ = (OR_k,a_ * P_1,a_ / (1 - P_1,a_ + OR_k,a_ * P_1,a_) (6)

P_k,a_ is the probability of being stunted in the riskier status (e.g., not receiving zinc for prevention). P_1,a_ is the probability of being stunted in the less risky status. OR_k,a_ is the odds ratio. The weighted sum of the probabilities will equal the overall probability of stunting as indicated by equation (7).

P_a,t_ = F_k,a,t_ * P_k,a_ + F_1,a,t_ * P_1,a_ (7)

P_a,t_ is the probability of stunting among children in age band “a” at time “t”. F_k,a,t_ and F_1,a,t_ are the fractions of the children in the age band who are in the risky status and the non-risky status respectively. At the baseline, Equations 6 and 7 are two equations in two unknowns that can be solved for the risk of stunting for the children in the non-risky status (P_1,a_ ) and for the children in the risky status (P_k,a_). After having solved these two equations, the probability of stunting can be calculated based on changing levels of the risky versus non-risky status using the values of P_1,a_ and P_1,k_. The independent percent reduction in stunting that would be caused by the reduction in the more risky status is calculated by equation 8.

R_k,t_ = ( P_a,t_ - P_a,0_) / P_a,0_ (8)

The following presents an example calculation with zinc for prevention. Suppose 25 percent of children receive zinc supplementation at the baseline 35 percent of children are stunted. F_k,a,t_ would be the percent of children who do not receive zinc supplementation or 75 percent. In LiST the default odds ratio of being stunted for children who are zinc supplemented relative to those who are not zinc supplemented is 1.18. If equations 6 and 7 are solved simultaneously then the percent of zinc supplemented children who are stunted is 32.2 and the percent of un-supplemented children who are stunted is 35.9.

If preventive zinc supplementation were the only intervention and the percent of children who are supplemented were to increase from 25 percent to 50 percent then the percent stunted would decline from 35 percent to 34.1 percent via equation 7 (0.50 x 0.322 + 0.50 x 0.359 = 0.341). And the percent decline in stunting would be 2.7 percent via equation 8 ( (0.350 – 0.341)/0.350 = 0.027).

This process would be replicated for all statuses or behaviors impacting stunting status. The overall reduction in stunting is calculated similar to the process described above in equations (1) and (2).

In LiST there are five factors that influence stunting:

• For neonatal children the percent who were born with IUGR;

• For post-neonatal children the percent who were stunted at the previous age band;

• Complementary feeding;

• Episodes of diarrhoea per year; and

• Zinc supplementation.

LiST includes two interventions related to feeding children. Complementary feeding is an intervention designed to address stunting. Supplementary feeding is an intervention designed to address wasting. The percent of children born IUGR is impacted by improvements in coverage of pregnant women protected via Intermittent Prevention Therapy (IPT) or sleeping under a bednet, balanced energy supplementation or multiple micronutrient supplementations. The percent reduction in children born IUGR is calculated with the strategy described in equations (1) and (2) with the percentage reduction in mortality replaced by the percentage reduction in children born with IUGR.

The impact of complementary feeding is described by odds ratios associated with four different states:

• Food secure with promotion;

• Food secure without promotion;

• Food insecure with promotion and supplementation; and

• Food insecure without promotion and supplementation.

Food secure populations are defined as those living above the poverty line. The isolated impact of complementary feeding is calculated with the strategy described by equations (6) through (8). A major difference is that equation 6 is replaced with three equations corresponding to three odds ratios defined relative to the food secure population with promotion, the least risky group. Equation 7 is replaced with an equation that has four terms on the right hand side corresponding to the four bulleted populations listed above. These four equations (three equations replacing equation 6 and the new equation 7) lead to a quartic equation that is solved analytically to obtain the baseline stunting probabilities for the food secure population with promotion, the food secure population without promotion, the food secure population with promotion and supplementation and the food secure population with neither promotion nor supplementation. The remainder of the calculations are the same as described above.

The impact of reduced diarrhoea incidence on stunting is modeled via an odds ratio that is denominated as an odds ratio that increases as the number of episodes of diarrhoea increases. The population averag odds ratio is the odds ratio of a single case raised to the power of the average number of cases of diarrhoea per year (*i*).

AO_k,a,t_ = (OR_k,a_)^i,t^ (10)

This formulation is converted into the approach of equations (6) through (8) by assuming that all children in the base year have “i” cases of diarrhoea and that reductions in diarrhoea incidence are achieved by moving a proportion of the children from having “i” cases of diarrhoea to having no diarrhoea at all. For example assume that there are on average 3 cases of diarrhoea per child in the baseline year and on average 2 cases of diarrhoea in the target year. This would be translated into 66.7 percent of the children having 3 cases of diarrhoea (analogous to F_a,k,t_) and 33.3 percent of the children having no diarrhoea (analogous to F_a,1,t_) in the target year.

Diarrhoea incidence is itself an intermediate outcome in LiST. It is affected by water and sanitation improvements. The water and sanitation interventions include improved water source within 30 minutes, use of a water connection in the home, improved excreta disposal (latrine/toilet), hand washing with soap and hygienic disposal of children’s stools. The impact of these interventions is calculated following the strategy described by equations (1) through (3) above where the percent reduction in mortality and the mortality rate are replaced by percent reduction in diarrhoea incidence and number of cases of diarrhoea per year.

Theoretically and empirically breastfeeding has an impact on diarrhoea incidence. Disentangling the direct nutritional impact of breastfeeding and the indirect impact of breastfeeding via reduced diarrhoea incidence is very difficult or impossible. Rather than attempt to disentangle these effects, LiST employs a combined effectiveness value for breastfeeding. This is described elsewhere in this article.

The impact of all interventions affecting stunting is combined as in equation 2 above by applying the percent reduction in stunting caused by a single intervention to the amount of stunting remaining after the impact of previous interventions has been included.

#### Wasting

Wasting is impacted by only one intervention, supplemental feeding, that is given only to severely wasted children (those less than three standard deviations below the international norm median for weight for height). Starting from a normally distributed population based on current levels of wasting, wasted children receiving supplemental feeding are shifted into different bands within the international standard distribution for weight for height for health children. These bands into which children are moved are:

• More than 1 standard deviation less than the median norm

• Between 1 and 2 standard deviations less than the median norm

• Between 2 and 3 standard deviations less than the median norm.

For some children supplemental feeding is not at all effective. They remain at below 3 standard deviations less than the median norm.

### Calculating the effects of intermediate health outcomes on mortality

The stunting calculations in the previous section determined the percent of children who are stunted or not stunted. Stunting is the percent of children who are less than two standard deviations below the international norm of height for age. Within LiST however the risks for mortality vis-à-vis stunting are defined in terms of the following four bands on the international normal distribution for height for age:

• More than 1 standard deviation less than the median norm

• Between 1 and 2 standard deviations less than the median norm

• Between 2 and 3 standard deviations less than the median norm

• Below 3 standard deviations less the median norm.

**Figure 3:**
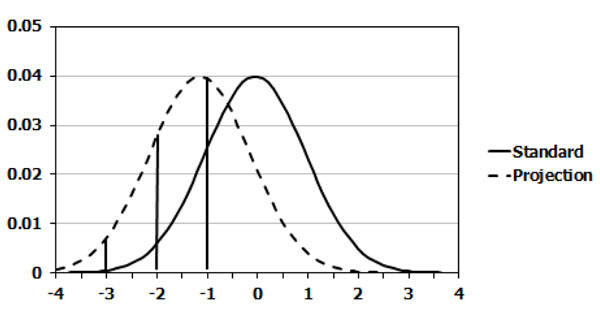
Illustration of the calculation of the percent of children who are < -3 SD, -3 to <-2 SD, -2 to -1 SDs and above -1 SDs for height to age relative to international standard

Immediately following is a description of how the stunting is translated into these four bands. LiST assumes that height for age is distributed normally with a standard deviation of one. Using the percent stunted or wasted, LiST calculates the percent of children who are at various points along the international standard curve for height for age and weight for height. Children are then assigned to one of the four categories based upon these calculations. Figure 2 shows the distribution of children in relation to the norm in an illustrative case for stunting. The curve on the right represents the international standard. The curve on the left represents the actual situation in the illustrative case. In the international standard the percent of children who are stunted (more than 2 standard deviations below the norm) is a relatively small proportion (2.5%) of the population in the left hand tail of the distribution. In the illustrative case, approximately 50 percent of the children are stunted. The vertical bars under the curve on the left show the points in the distribution that correspond to -1 SD, -2 SD and -3 SD in the international standard. From these curve LiST calculates the percent of children falling into the four key categories: < -3 SD, -3 to <-2 SD, -2 to -1 SDs and -1 - 0 SDs. Illustrations of these calculations can be found in figure 3.

Each of the height for age or weight for height statuses is associated with a risk of cause specific mortality relative to normal height for age and weight for height. For example children aged 1-5 months who are less than three standard deviations below the international norm for height for age are 4.6 times more likely to die of diarrhoea than are children who are greater than one standard deviation less than the international median norm.

Percent reductions in cause specific mortality are established by first calculating the average relative risk of mortality relative to the reference. “Reference” in the next few paragraphs refers to greater than one standard deviation less than the international median norm. The average relative risk (ARR) for a specific cause of death (*j*) and a particular age (*a*) at time *t* is the sum across all categories (*s*) of height for age or weight for height of the percentage of children in that category (Z) multiplied by the relative risk (RR) of death for children in that category. The reference category (children of normal height for age or weight for height) has a relative risk of 1.00.

ARR_a,ji,t_ = ∑_s_ Z_s,a,t_ x RR_s,a,j, t_ (11)

The percent reduction in mortality is then calculated as 1 minus the ratio of the average relative risk at time *t* to the average relative risk in the base year.

R_a,i,t_ = 1 – ARR_a,i,t_ / ARR_a,i,0_ (12)

Note that this equation represents the reduction in mortality that would occur if only reductions in stunting were impacting mortality. In the general case this reduction is combined with reductions in mortality due to other interventions as if stunting were also an intervention. The shares of mortality reduction accounted for by interventions that cause reductions in stunting are established recursively via the share of mortality accounted for by reductions in stunting and then by the shares of stunting reduction accounted for by the interventions which reduce stunting.

The impact of reduced wasting on mortality is handled exactly the same as stunting. Decreased wasting leads to a shift of the distribution of weight for height scores.

#### IUGR impact on neonatal mortality

The direct impact of IUGR on mortality is calculated in a similar manner via reductions in relative risk. The major difference is that instead of four categories of stunting only two states, IUGR or no IUGR, are included.

#### Impact of improved breastfeeding on mortality

In the original version of LiST breastfeeding was an intermediate outcome that improved as a result of scaling up breastfeeding promotion. In the current version of LiST, breastfeeding may be an intermediate outcome or it may be entered directly by the user as if it were an intervention. In reality breastfeeding is a risk factor, but for calculation purposes it is handled as if it were an intervention. Breastfeeding is modeled via four categories: exclusive breastfeeding, predominant breastfeeding, partial breastfeeding and no breastfeeding. These categories are disaggregated by age.

The impact of breastfeeding promotion is modeled via odds ratios. The calculation process is similar to that used in equations 6 - 8. The major difference is that the odds ratios are structured as the odds of improved breastfeeding without promotion versus the odds with promotion.

Mortality reductions associated with improved breastfeeding are calculated in the same way as mortality reductions are calculated for reduced stunting. However, instead of children being in categories falling along a normal curve, risk is defined by the type of breastfeeding.

### Illustrations of the complexity of LiST

**Figure 4:**
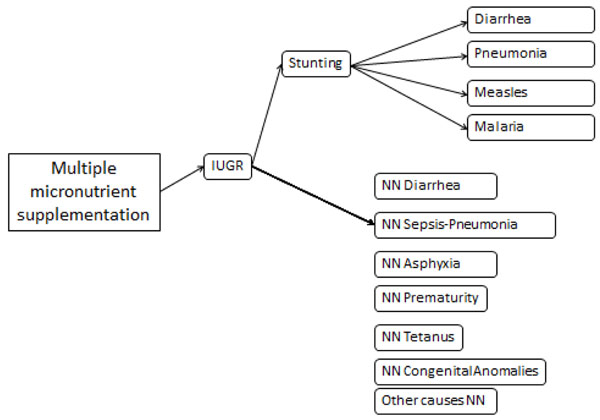
LiST calculation of mortality reduction due to scaling up multiple micronutrient supplementation

This paper does not discuss all aspects of LiST as that would take too much space. Figures 1 and 4 exhibit the complexity of LiST from two directions: from the standpoint of a cause of death (postneonatal diarrhoea) and a single intervention (multiple micronutrient supplementation). Figure 4 is conceptually shaped like a funnel with multiple interventions funneling in and at the end generating a reduction in postneonatal diarrhoea mortality. Some interventions such as Zinc treatment directly impacts diarrhoeal mortality. Other interventions such as complementary feeding impact diarrhoeal deaths indirectly via a nutritional status such as stunting. And a third group of interventions affect diarrhoea mortality directly and indirectly. An example of this would be hand washing which impacts diarrhoea incidence which in turn impacts stunting and then diarrhoea mortality.

Figure 4 flips the funnel over to show how a single intervention can impact multiple causes of death like a shotgun blast. Multiple micronutrient supplementation impacts intra-uterine growth retardation (IUGR). This in turn directly impacts several neonatal causes of death. IUGR also impacts subsequent stunting. Reductions in stunting then impacts several postneonatal causes of death.

## Model parameters and baseline data inputs

The key parameters in the calculation of intervention effects on mortality include current health status indicators, the distribution of deaths by cause, the effectiveness of each intervention and current and future intervention coverage. The key parameters and sources of baseline data are shown in Table 2. National health surveys such as the Demographic and Health Surveys (DHS) and the Multiple Indicator Cluster Surveys (MICS) provide most of the data on current mortality rates, the prevalence of stunting and wasting, and the current coverage of interventions. Other health status indicators are drawn from WHO databases. Estimates of intervention effectiveness have been developed by the Child Health Epidemiology Reference Group (CHERG). Most of that work has been published previously. [6]

**Table 2:** Key Parameters in LiST and Sources of Baseline Information

Parameter	Source
**Health Status Indicators**	
Neonatal, infant and under five mortality rate	United Nations Estimates
Distribution of neonatal and post-neonatal deaths by cause	CHERG
Whether or not the population of interest is Vitamin A deficient and/or zinc deficient	CHERG
Percent of women exposed to falciparum	Guerra et al.[8]
Percent of newborns with IUGR	DHS, MICS, UNICEF, WHO
Percent of children severely wasted by age	DHS, MICS, WHO (http://www.who.int/nutgrowthdb/database/en)
Percent of children stunted by age	DHS, MICS, WHO (http://www.who.int/nutgrowthdb/database/en)
Incidence of diarrhoea by age	Boschi et al. [9]
Percent of pregnancies ending with spontaneous abortion	WHO
Percentage of the population living below the poverty line	Human Development Report, UNDP
**Intervention Effectiveness**	
Effectiveness of each intervention against each cause of death	CHERG
Affected fraction (fraction of deaths from a specific cause potentially addressed by each intervention)	CHERG
Effectiveness of nutrition-related interventions against IUGR, stunting, wasting and diarrhoea incidence	CHERG
Effectiveness of breastfeeding promotion on breastfeeding practices	CHERG
**Coverage**	
Current coverage of each intervention	DHS, MICS, UNICEF, WHO, JMP

## Using LiST

LiST is a component of the Spectrum program for policy modeling. Spectrum may be downloaded from several web servers including http://www.jhsph.edu/dept/ih/IIP/list/. Detailed instructions on installation are at this site. The program contains all the parameter values described above for 77 maternal and child interventions, including current intervention coverage for most countries. If the model is run without any changes the coverage of all interventions will remain constant at the current level and, as a result, mortality rates will also remain constant.

LiST can be used to explore the effects of alternate strategies by scaling up coverage of selected interventions over time. LiST will calculate the expected change in mortality as a result of the changes in coverage. The number of possible strategies that can be examined using LiST is quite large.

LiST is designed to encourage a strategic approach to strategy selection. The first step is to examine the mortality rates to see when most mortality happens. If mortality is concentrated in the neonatal period, then interventions that reduce neonatal mortality should be examined close. A second step is to examine the distribution of deaths by cause. If one or two causes of death are responsible for most deaths then, interventions that are effective against those causes of death are likely candidates. The most effective strategies will be those that scale up interventions that have large effects and those that currently have low coverage.

## Model outputs

LiST calculates the effects of health interventions on neonatal, child and maternal mortality. The key outputs for each type of mortality are:

• the mortality rates (neonatal, infant, under five and maternal mortality rates)

• the number of deaths

• the number of still births

• the number of deaths by cause

• the number of deaths averted

• the number of deaths averted by cause

• the number of deaths averted by intervention

LiST also provides outputs related to nutrition including the percentage of children severely wasted, the percentage stunted, average height/length, breastfeeding prevalence, diarrhoea incidence and the prevalence of IUGR. All indicators are available for each year of the projection.

## Conclusions

This paper restricted its discussion to the child mortality aspects of The Lives Saved Tool (LiST). LiST also includes the capacity to calculate reductions in maternal mortality and reductions in still births. The calculations for these run in parallel to those for child mortality. All interventions for child mortality, maternal mortality and still births are scaled up in the same editors assuring consistent estimates of all outcomes. The calculations for maternal mortality and still births do not require turning on special features. They are calculated automatically when LiST is implemented.

The Lives Saved Tool, LiST, is intended to support national planning to improve maternal and child health. It summarizes a vast impact assessment literature by providing consensus estimates of the effectiveness of health interventions in a tool that facilitates the application of this information to any national context. LiST provides planners and policy makers with a tool to examine the potential impact of alternative strategies to reduce mortality. It is intended to support the strategic analysis of alternatives by displaying the cause of death structure and producing output showing not only the total impact on mortality but also the contribution of each intervention to the total impact.

There are limitations to LiST. It can be difficult to ensure that data on coverage of interventions refers to interventions that are similar to those in the impact literature. For some interventions estimates of effectiveness may rely on a small number of studies. The current version of LiST does not address these uncertainties although we expect to add this feature to future versions. The current version does not consider cost, but work is underway to add costing the model. Future versions will also include the ability to add interventions, for example a new vaccine, into the model..

LiST is readily available to anyone who wants to use it. It contains data bases to facilitate use. Most of the key assumptions in LiST are addressed in published articles in this supplement and earlier publications. [6] Using LiST properly requires an investment in time to fully understand the epidemiological context of any particular country and to construct realistic scenarios detailing increases in coverage for key interventions. However, in most cases this is time well spent if it can lead to more strategic plans for scaling up key health interventions and averting many unnecessary deaths.

## Competing interests

The authors have no competing interests.

## Authors' contributions

WW developed many of the methods described here and wrote the first draft of this article. JS revised the first draft extensively. RM has been responsible for implementing these methods in the LiST computer program.

## Supplementary Material

Additional file 1 Interventions in LiST organized by when the intervention occurs. Click here for file
