# Supplementary material for: Methods used in the Lives Saved Tool (LiST)
Source: BMC Public Health. 2011 Apr 13;11(Suppl 3):S32. doi: 10.1186/1471-2458-11-S3-S32 (PMC3231906; doi:10.1186/1471-2458-11-S3-S32)
Supplement: Additional file 1 — Interventions in LiST organized by when the intervention occurs. [file 1471-2458-11-S3-S32-S1.docx]

**Additional File 1: Interventions in LiST Organized by When the Intervention Occurs**

**Periconceptual Period**

Contraception

Folic acid supplementation or fortification

Abortion services

Safe abortion services

Case management

Post abortion case management basic EMOC level

Post abortion case management comprehensive EMOC level

Ectopic case management basic EMOC level

Ectopic case management comprehensive EMOC level

**Pregnancy**

Antenatal care

Syphilis detection and treatment

Calcium supplementation

Multiple micronutrient supplementation

Pregnant women protected via IPT or sleeping under an ITN

Tetanus toxoid

Balanced energy supplementation

Case management during pregnancy

Identification of women at risk of hypertensive diseases of pregnancy, and treatment with aspirin, if needed

Hypertensive disease case management (facility)

MgSO4 management of pre-eclampsia

Diabetes screening and management

Case management of malaria (clinic)

Case management of malaria (hospital)

Screening for fetal growth restriction and appropriate management

PMTCT

**Childbirth**

Basic data (for calculating coverage of delivery interventions)

Institutional delivery (clinic and hospital)

Skilled birth attendance (SBA)

At onset of labour or risk of onset

Antenatal corticosteroids for preterm labor

Antibiotics for pPRoM

Institutional delivery (clinic and hospital)

Essential care for all women and immediate essential newborn care

Basic emergency obstetric care (clinic)

Comprehensive emergency obstetric care

Induction of labour to prevent births at or beyond 41 completed weeks

Active management of the 3rd stage of labour

MgSO4 management of eclampsia

Neonatal resuscitation (institutional)

Home delivery

Clean practices and immediate essential newborn care (home)

Neonatal resuscitation (home)

Unassisted delivery

**Breastfeeding**

Breastfeeding promotion

**Preventive Interventions after Birth**

Preventive postnatal care (healthy practices and illness detection)

Complementary feeding--education only

Complementary feeding--supplementation and education

Use of improved water source within 30 minutes

Use of water connection in the home

Improved excreta disposal (latrine/toilet)

Hand washing with soap

Hygienic disposal of children's stools

Insecticide treated materials or indoor residual spraying

Vitamin A for prevention

Zinc for prevention

**Vaccines**

Rotavirus vaccine

Measles vaccine

Hib vaccine

Pneumococcal vaccine

DPT vaccination

Polio vaccine

BCG vaccine

**Curative Interventions after Birth**

Sepsis case management - basic

Sepsis case management - comprehensive

Kangaroo mother care

Oral antibiotics: case management of severe neonatal infection

Injectable antibiotics: case management of severe neonatal infection

Full supportive care: case management of severe neonatal infection

ORS

Antibiotics for dysentery

Zinc for treatment

Case management of pneumonia (oral antibiotics)

Vitamin A for measles treatment

Antimalarials

Therapeutic feeding

Cotrimoxazole

ART
